# 4-Methyl­benzaldehyde thio­semi­carbazone

**DOI:** 10.1107/S1600536809033297

**Published:** 2009-08-26

**Authors:** Jian Zhang, Hao Geng, Ling-hua Zhuang, Guo-wei Wang

**Affiliations:** a Department of Light Chemical Engineering, College of Food Science and Light Engineering, Nanjing University of Technology, Nanjing 210009, People’s Republic of China

## Abstract

The title compound, C_9_H_11_N_3_S, was prepared by reacting 4-methyl­benzaldehyde with thio­semicarbazide. An intra­molecular N—H⋯N hydrogen bond helps to establish the observed mol­ecular conformation. The crystal packing is realized by inter­molecular N—H⋯S hydrogen bonds.

## Related literature

For general background to thio­semicarbazone compounds, see: Casas *et al.* (2000 ▶); Tarafder *et al.* (2000 ▶); Ferrari *et al.* (2000 ▶); Deschamps *et al.* (2003 ▶); Maccioni *et al.*(2003 ▶); Chimenti *et al.* (2007 ▶); Zhang *et al.* (2009 ▶). For bond-length data, see: Allen *et al.* (1987 ▶).
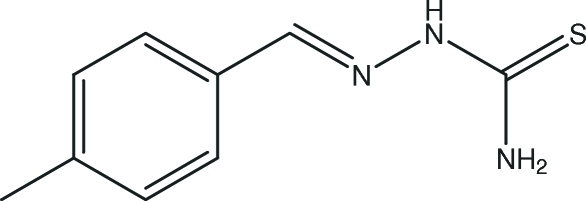


## Experimental

### 

#### Crystal data

C_9_H_11_N_3_S
*M* *_r_* = 193.27
Monoclinic, 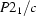

*a* = 13.234 (3) Å
*b* = 8.221 (2) Å
*c* = 10.311 (2) Å
β = 111.15 (3)°
*V* = 1046.2 (4) Å^3^
*Z* = 4
Mo *K*α radiation
μ = 0.27 mm^−1^
*T* = 293 K
0.30 × 0.20 × 0.10 mm

#### Data collection

Enraf–Nonius CAD-4 diffractometer
Absorption correction: ψ scan (North *et al.*, 1968 ▶) *T*_min_ = 0.924, *T*_max_ = 0.974
1982 measured reflections
1898 independent reflections
1322 reflections with *I* > 2σ(*I*)
*R*_int_ = 0.032
3 standard reflections every 200 reflections intensity decay: 9%

#### Refinement

*R*[*F* ^2^ > 2σ(*F* ^2^)] = 0.052
*wR*(*F* ^2^) = 0.146
*S* = 1.00
1898 reflections
120 parameters
H-atom parameters constrained
Δρ_max_ = 0.26 e Å^−3^
Δρ_min_ = −0.20 e Å^−3^

### 

Data collection: *CAD-4 Software* (Enraf–Nonius, 1989 ▶); cell refinement: *CAD-4 Software*; data reduction: *XCAD4* (Harms & Wocadlo, 1995 ▶); program(s) used to solve structure: *SHELXS97* (Sheldrick, 2008 ▶); program(s) used to refine structure: *SHELXL97* (Sheldrick, 2008 ▶); molecular graphics: *SHELXTL* (Sheldrick, 2008 ▶); software used to prepare material for publication: *SHELXTL*.

## Supplementary Material

Crystal structure: contains datablocks global, I. DOI: 10.1107/S1600536809033297/im2136sup1.cif

Structure factors: contains datablocks I. DOI: 10.1107/S1600536809033297/im2136Isup2.hkl

Additional supplementary materials:  crystallographic information; 3D view; checkCIF report

## Figures and Tables

**Table 1:** Hydrogen-bond geometry (Å, °)

*D*—H⋯*A*	*D*—H	H⋯*A*	*D*⋯*A*	*D*—H⋯*A*
N2—H2*A*⋯S^i^	0.86	2.54	3.389 (3)	168
N3—H3*B*⋯S^ii^	0.86	2.61	3.395 (3)	153
N3—H3*A*⋯N1	0.86	2.29	2.641 (4)	105

Symmetry codes: (i) 
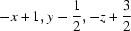
; (ii) 
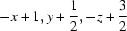
.

**Fig. 1.**
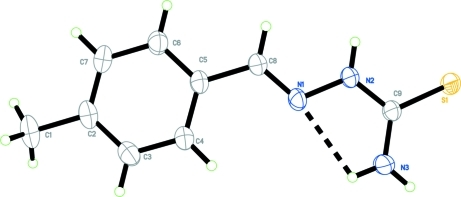
Molecular structure of (I) showing the atom-numbering scheme and 30% displacement ellipsoids. The intramolecular hydrogen bond is shown as dashed lines.

**Fig. 2.**
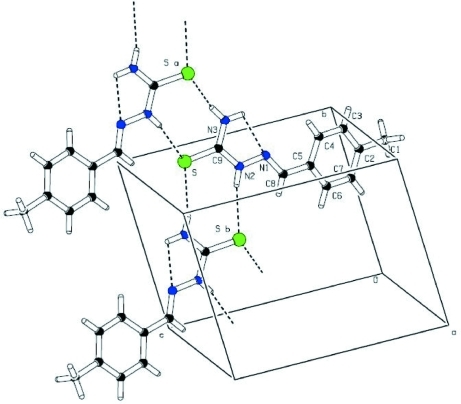
Crystal packing of (I). Hydrogen bonds are drawn as dashed lines.

## supplementary crystallographic information

### Comment

Thiosemicarbazones constitute an important class of N,*S* donor ligands due to their ability to react with a wide range of metals (Casas *et al.*, 2000). Thiosemicarbazones exhibit various biological activities and have therefore attracted considerable pharmaceutical interest (Maccioni *et al.*, 2003; Ferrari *et al.*, 2000). They have been evaluated as antiviral, antibacterial and anticancer therapeutics. Thiosemicarbazones belong to a large group of thiourea derivatives, whose biological activities are a function of the parent aldehyde or ketone moiety (Chimenti *et al.*, 2007). Schiff bases in general show potential as antimicrobial and anticancer agents (Tarafder *et al.*, 2000; Deschamps *et al.*, 2003) and have therefore envisaged biochemical and pharmacological applications. We are focusing our synthetic and structural studies on new products of thiazole Schiff bases from thiosemicarbazones (Zhang *et al.*, 2009). We herein report the crystal structure of the title compound (I).

The atom-numbering scheme of (I) is shown in Fig.1, and all bond lengths are within normal ranges (Allen *et al.*, 1987).

The sulfur atom and the hydrazine nitrogen N1 are in *trans* position with respect to the C9–N2 bond. The molecular conformation is determined by a strong intramolecular hydrogen bond N3–H3A···N1(2.641 (3) A°).

The planar phenyl ring A (C2/C3/C4/C5/C6/C7, r.m.s. deviation 0.0115 (1) Å) and the pseudo five-membered ring B(N1/N2/C9/N3/H3A, r.m.s. deviation 0.035 (1) Å that is formed by the N3—H3A···N1 hydrogen bond enclose a dihedral angle of 14 (1)°. The dihedral angle between the thiourea group (N1/N2/C9/S) and the phenyl ring measures to 17 (2)°.

The crystal packing is realized by intermolecular N—H···S hydrogen bonds (Table 1, Fig. 1 and Fig.2).

### Experimental

A mixture of 4-methyl-benzaldehyde (1.20 g, 0.01 mol) and hydrazinecarbothioamide (0.91 g, 0.01 mol) in 20 ml of absolute methanol was refluxed for about 3 h. After cooling, the precipitated solid separated was filtered and recrystallized from ethyl acetate. Crystals of (I) suitable for X-ray diffraction were obtained by slow evaporation of ethyl acetate at room temperature. ^1^H NMR (DMSO, δ, p.p.m.) 11.39 (s, 1 H), 8.17 (s, 1 H), 8.02 (s,2 H), 7.42 (m, 1 H), 7.30 (t, 2 H), 6.99 (t,1 H), 1.79 (t, 3 H).

### Refinement

All H atoms were positioned geometrically, with C—H = 0.93 Å and N—H = 0.86 °, and constrained to ride on their parent atoms, with *U*_iso_(H) = *xU*_eq_(C), where *x*= 1.5 for methyl H and *x* = 1.2 for methylene H atoms.

### Crystal data

**Table:**

C_9_H_11_N_3_S	*F*(000) = 408
*M**_r_* = 193.27	*D*_x_ = 1.227 Mg m^−^^3^
Monoclinic, *P*2_1_/*c*	Mo *K*α radiation, λ = 0.71073 Å
Hall symbol: -P 2ybc	Cell parameters from 27 reflections
*a* = 13.234 (3) Å	θ = 1–25°
*b* = 8.221 (2) Å	µ = 0.27 mm^−^^1^
*c* = 10.311 (2) Å	*T* = 293 K
β = 111.15 (3)°	Block, colorless
*V* = 1046.2 (4) Å^3^	0.30 × 0.20 × 0.10 mm
*Z* = 4	

### Data collection

**Table:**

Enraf–Nonius CAD-4 diffractometer	1322 reflections with *I* > 2σ(*I*)
Radiation source: fine-focus sealed tube	*R*_int_ = 0.032
graphite	θ_max_ = 25.3°, θ_min_ = 1.7°
ω/2θ scans	*h* = −15→0
Absorption correction: ψ scan (North *et al.*, 1968)	*k* = 0→9
*T*_min_ = 0.924, *T*_max_ = 0.974	*l* = −11→12
1982 measured reflections	3 standard reflections every 200 reflections
1898 independent reflections	intensity decay: 9%

### Refinement

**Table:**

Refinement on *F*^2^	Secondary atom site location: difference Fourier map
Least-squares matrix: full	Hydrogen site location: inferred from neighbouring sites
*R*[*F*^2^ > 2σ(*F*^2^)] = 0.052	H-atom parameters constrained
*wR*(*F*^2^) = 0.146	*w* = 1/[σ^2^(*F*_o_^2^) + (0.07*P*)^2^ + 0.38*P*] where *P* = (*F*_o_^2^ + 2*F*_c_^2^)/3
*S* = 1.00	(Δ/σ)_max_ < 0.001
1898 reflections	Δρ_max_ = 0.26 e Å^−^^3^
120 parameters	Δρ_min_ = −0.20 e Å^−^^3^
0 restraints	Extinction correction: *SHELXL97* (Sheldrick, 2008), Fc^*^=kFc[1+0.001xFc^2^λ^3^/sin(2θ)]^-1/4^
Primary atom site location: structure-invariant direct methods	Extinction coefficient: 0.034 (4)

### Special details

**Table:**

Geometry. All e.s.d.'s (except the e.s.d. in the dihedral angle between two l.s. planes) are estimated using the full covariance matrix. The cell e.s.d.'s are taken into account individually in the estimation of e.s.d.'s in distances, angles and torsion angles; correlations between e.s.d.'s in cell parameters are only used when they are defined by crystal symmetry. An approximate (isotropic) treatment of cell e.s.d.'s is used for estimating e.s.d.'s involving l.s. planes.
Refinement. Refinement of *F*^2^ against ALL reflections. The weighted *R*-factor *wR* and goodness of fit *S* are based on *F*^2^, conventional *R*-factors *R* are based on *F*, with *F* set to zero for negative *F*^2^. The threshold expression of *F*^2^ > σ(*F*^2^) is used only for calculating *R*-factors(gt) *etc*. and is not relevant to the choice of reflections for refinement. *R*-factors based on *F*^2^ are statistically about twice as large as those based on *F*, and *R*- factors based on ALL data will be even larger.

### Fractional atomic coordinates and isotropic or equivalent isotropic displacement parameters (Å^2^)

**Table:**

	*x*	*y*	*z*	*U*_iso_*/*U*_eq_	
S	0.48277 (7)	0.09017 (9)	0.81196 (7)	0.0534 (3)	
N1	0.34064 (18)	−0.0455 (3)	0.4236 (2)	0.0438 (6)	
C1	−0.0005 (3)	−0.2600 (6)	−0.1894 (3)	0.0788 (12)	
H1B	−0.0374	−0.3614	−0.1934	0.118*	
H1C	0.0324	−0.2593	−0.2585	0.118*	
H1D	−0.0514	−0.1721	−0.2067	0.118*	
N2	0.40264 (19)	−0.0458 (3)	0.5647 (2)	0.0456 (6)	
H2A	0.4275	−0.1358	0.6065	0.055*	
C2	0.0863 (3)	−0.2397 (5)	−0.0467 (3)	0.0560 (9)	
N3	0.3955 (2)	0.2299 (3)	0.5633 (3)	0.0630 (8)	
H3A	0.3650	0.2263	0.4743	0.076*	
H3B	0.4078	0.3222	0.6054	0.076*	
C3	0.1264 (3)	−0.0877 (4)	0.0043 (3)	0.0614 (9)	
H3C	0.1006	0.0034	−0.0513	0.074*	
C4	0.2041 (3)	−0.0681 (4)	0.1365 (3)	0.0553 (8)	
H4A	0.2302	0.0352	0.1678	0.066*	
C5	0.2429 (2)	−0.2018 (4)	0.2222 (3)	0.0445 (7)	
C6	0.2060 (2)	−0.3553 (4)	0.1705 (3)	0.0521 (8)	
H6A	0.2329	−0.4465	0.2255	0.063*	
C7	0.1289 (3)	−0.3741 (4)	0.0370 (3)	0.0565 (9)	
H7A	0.1056	−0.4779	0.0036	0.068*	
C8	0.3157 (2)	−0.1842 (4)	0.3664 (3)	0.0452 (7)	
H8A	0.3446	−0.2770	0.4180	0.054*	
C9	0.4235 (2)	0.0939 (3)	0.6353 (3)	0.0420 (7)	

### Atomic displacement parameters (Å^2^)

**Table:**

	*U*^11^	*U*^22^	*U*^33^	*U*^12^	*U*^13^	*U*^23^
S	0.0798 (6)	0.0472 (5)	0.0282 (4)	−0.0056 (4)	0.0134 (4)	−0.0039 (3)
N1	0.0512 (15)	0.0501 (15)	0.0282 (11)	−0.0033 (11)	0.0121 (10)	−0.0016 (10)
C1	0.071 (2)	0.116 (3)	0.0399 (18)	−0.014 (2)	0.0086 (17)	−0.015 (2)
N2	0.0603 (16)	0.0443 (14)	0.0268 (11)	−0.0011 (12)	0.0094 (11)	−0.0010 (10)
C2	0.0497 (19)	0.081 (2)	0.0362 (16)	−0.0065 (17)	0.0143 (14)	−0.0080 (16)
N3	0.100 (2)	0.0425 (15)	0.0339 (13)	0.0016 (14)	0.0082 (14)	−0.0005 (11)
C3	0.068 (2)	0.067 (2)	0.0419 (17)	−0.0007 (18)	0.0119 (16)	0.0070 (16)
C4	0.064 (2)	0.055 (2)	0.0386 (16)	−0.0061 (16)	0.0082 (14)	−0.0039 (14)
C5	0.0452 (17)	0.0530 (18)	0.0342 (14)	−0.0006 (14)	0.0131 (12)	−0.0056 (13)
C6	0.0556 (19)	0.0562 (19)	0.0423 (17)	−0.0015 (15)	0.0151 (14)	−0.0060 (15)
C7	0.057 (2)	0.066 (2)	0.0451 (17)	−0.0113 (16)	0.0169 (15)	−0.0190 (16)
C8	0.0516 (18)	0.0476 (18)	0.0332 (14)	0.0002 (14)	0.0115 (13)	−0.0019 (13)
C9	0.0492 (17)	0.0438 (16)	0.0320 (14)	−0.0015 (13)	0.0135 (12)	−0.0018 (13)

### Geometric parameters (Å, °)

**Table:**

S—C9	1.704 (3)	N3—H3A	0.8600
N1—C8	1.271 (4)	N3—H3B	0.8600
N1—N2	1.389 (3)	C3—C4	1.390 (4)
C1—C2	1.514 (4)	C3—H3C	0.9300
C1—H1B	0.9600	C4—C5	1.387 (4)
C1—H1C	0.9600	C4—H4A	0.9300
C1—H1D	0.9600	C5—C6	1.388 (4)
N2—C9	1.334 (3)	C5—C8	1.458 (4)
N2—H2A	0.8600	C6—C7	1.395 (4)
C2—C3	1.385 (5)	C6—H6A	0.9300
C2—C7	1.390 (5)	C7—H7A	0.9300
N3—C9	1.319 (3)	C8—H8A	0.9300
			
C8—N1—N2	116.1 (2)	C5—C4—C3	120.4 (3)
C2—C1—H1B	109.5	C5—C4—H4A	119.8
C2—C1—H1C	109.5	C3—C4—H4A	119.8
H1B—C1—H1C	109.5	C4—C5—C6	118.5 (3)
C2—C1—H1D	109.5	C4—C5—C8	121.9 (3)
H1B—C1—H1D	109.5	C6—C5—C8	119.5 (3)
H1C—C1—H1D	109.5	C5—C6—C7	120.7 (3)
C9—N2—N1	119.9 (2)	C5—C6—H6A	119.7
C9—N2—H2A	120.1	C7—C6—H6A	119.7
N1—N2—H2A	120.1	C2—C7—C6	120.8 (3)
C3—C2—C7	117.9 (3)	C2—C7—H7A	119.6
C3—C2—C1	121.4 (3)	C6—C7—H7A	119.6
C7—C2—C1	120.8 (3)	N1—C8—C5	121.8 (3)
C9—N3—H3A	120.0	N1—C8—H8A	119.1
C9—N3—H3B	120.0	C5—C8—H8A	119.1
H3A—N3—H3B	120.0	N3—C9—N2	117.5 (2)
C2—C3—C4	121.6 (3)	N3—C9—S	123.0 (2)
C2—C3—H3C	119.2	N2—C9—S	119.5 (2)
C4—C3—H3C	119.2		
			
C8—N1—N2—C9	−173.6 (3)	C3—C2—C7—C6	2.8 (5)
C7—C2—C3—C4	−2.1 (5)	C1—C2—C7—C6	−177.7 (3)
C1—C2—C3—C4	178.4 (3)	C5—C6—C7—C2	−0.8 (5)
C2—C3—C4—C5	−0.7 (5)	N2—N1—C8—C5	174.4 (2)
C3—C4—C5—C6	2.8 (5)	C4—C5—C8—N1	5.3 (5)
C3—C4—C5—C8	−173.4 (3)	C6—C5—C8—N1	−170.9 (3)
C4—C5—C6—C7	−2.0 (5)	N1—N2—C9—N3	−8.5 (4)
C8—C5—C6—C7	174.2 (3)	N1—N2—C9—S	170.71 (19)

### Hydrogen-bond geometry (Å, °)

**Table:**

*D*—H···*A*	*D*—H	H···*A*	*D*···*A*	*D*—H···*A*
N3—H3A···N1	0.86	2.29	2.641 (4)	105
N2—H2A···S^i^	0.86	2.54	3.389 (3)	168
N3—H3B···S^ii^	0.86	2.61	3.395 (3)	153

Symmetry codes: (i) −*x*+1, *y*−1/2, −*z*+3/2; (ii) −*x*+1, *y*+1/2, −*z*+3/2.
